# Lineage specific recombination rates and microevolution in *Listeria monocytogenes*

**DOI:** 10.1186/1471-2148-8-277

**Published:** 2008-10-08

**Authors:** Henk C den Bakker, Xavier Didelot, Esther D Fortes, Kendra K Nightingale, Martin Wiedmann

**Affiliations:** 1 Department of Food Science, Cornell University, Ithaca, NY, USA; 2 Department of Statistics, University of Warwick, Coventry, CV4 7AL, UK; 3 Department of Animal Science, Colorado State University, Fort Collins, CO, USA

## Abstract

**Background:**

The bacterium *Listeria monocytogenes *is a saprotroph as well as an opportunistic human foodborne pathogen, which has previously been shown to consist of at least two widespread lineages (termed lineages I and II) and an uncommon lineage (lineage III). While some *L. monocytogenes *strains show evidence for considerable diversification by homologous recombination, our understanding of the contribution of recombination to *L. monocytogenes *evolution is still limited. We therefore used STRUCTURE and ClonalFrame, two programs that model the effect of recombination, to make inferences about the population structure and different aspects of the recombination process in *L. monocytogenes*. Analyses were performed using sequences for seven loci (including the house-keeping genes *gap*, *prs*, *purM *and *ribC*, the stress response gene *sigB*, and the virulence genes *actA *and *inlA*) for 195 *L. monocytogenes *isolates.

**Results:**

Sequence analyses with ClonalFrame and the Sawyer's test showed that recombination is more prevalent in lineage II than lineage I and is most frequent in two house-keeping genes (*ribC *and *purM*) and the two virulence genes (*actA *and *inlA*). The relative occurrence of recombination versus point mutation is about six times higher in lineage II than in lineage I, which causes a higher genetic variability in lineage II. Unlike lineage I, lineage II represents a genetically heterogeneous population with a relatively high proportion (30% average) of genetic material imported from external sources. Phylograms, constructed with correcting for recombination, as well as Tajima's D data suggest that both lineages I and II have suffered a population bottleneck.

**Conclusion:**

Our study shows that evolutionary lineages within a single bacterial species can differ considerably in the relative contributions of recombination to genetic diversification. Accounting for recombination in phylogenetic studies is critical, and new evolutionary models that account for the possibility of changes in the rate of recombination would be required. While previous studies suggested that only *L. monocytogenes *lineage I has experienced a recent bottleneck, our analyses clearly show that lineage II experienced a bottleneck at about the same time, which was subsequently obscured by abundant homologous recombination after the lineage II bottleneck. While lineage I and lineage II should be considered separate species from an evolutionary viewpoint, maintaining single species name may be warranted since both lineages cause the same type of human disease.

## Background

*Listeria monocytogenes *is a common bacterium that can be found in a large number of different natural and man-made habitats. While it is well adapted to a saprotrophic lifestyle, it can cause severe invasive disease in humans and a wide range of animals with manifestations including septicemia, encephalitis and late-term abortion. Human listeriosis represents a foodborne infection that predominantly affects the elderly, immunocompromised individuals, and pregnant women [1]. Based on most molecular subtyping methods, *L. monocytogenes *isolates can be subdivided into three main lineages, termed lineages I, II, and III [2]. Lineage I strains are significantly overrepresented among human listeriosis cases [3], while lineage II strains have a higher prevalence among isolates from environmental samples, foods, and animal listeriosis cases [2,4]. Lineage III strains are rare and mainly associated with animals [5]. Recent evidence suggests that isolates originally classified into lineage III represent a polyphyletic group [6] that includes at least three different lineages (lineage IIIA, IIIB and IIIC), which all contain serotype 4a, 4c, and atypical serotype 4b isolates.

Recombination plays an important role in the evolution of most bacterial species [7-9] and has often been presented as the equivalent of sex in eukaryotes. It constitutes a means for the rapid introduction of new genetic material into the genome, thus providing a much more rapid mode of evolution than point mutations [10,11]. Abundant recombination has also been associated with increased pathogenicity of selected bacterial strains [9]. Recombination poses a number of challenges for the evolutionary biologist as it blurs species boundaries and hampers proper inference reconstruction of the evolution of recombining organisms. For example, a comparative genomic study of *Escherichia coli *and *Salmonella enterica *[12] demonstrated that certain regions of the genome can be involved in inter-species recombination, even tens of millions of years after divergence. Recombination does not only violate the assumption of strict bifurcation of most phylogenetic algorithms [13], but also makes it difficult to infer the mutation rate and the age of the most recent common ancestor of groups of recombining organisms [14].

The goal of this study was to improve our understanding of the evolution and population structure of *L. monocytogenes*, with a particular focus on the role of recombination in lineage I and II. As previous studies [2,15,16] have suggested that there is a considerable amount of homologous recombination in the genome of at least some *L. monocytogenes*, we used STRUCTURE [17] and ClonalFrame [14], two programs that model the effect of recombination in genetic diversification, to draw inferences about the population structure and different aspects of the recombination process in *L. monocytogenes*.

## Methods

### *L. monocytogenes *isolates

Our study used multilocus sequence typing data for 195 *L. monocytogenes *isolates. A total of 184 isolates had been obtained from human clinical cases (n = 60), foods (n = 30) and animal clinical cases (n = 30) (as previously described by Nightingale *et al*. [2]) as well as natural and urban environments (n = 43) [4] and ruminants without clinical symptoms and farm environments (n = 21) [18]. All of these isolates were obtained from sources within New York State between 1999 and 2003. DNA sequences for all but the 21 isolates from ruminants without clinical symptoms and farm environments have previously been reported [18]. All isolates had previously been characterized by automated ribotyping and classified into lineages I, II, and III based on ribotype data. Because only four lineage III isolates were found among these 184 isolates we also included sequence data for 11 additional lineage III isolates representing lineage IIIA, IIIB and IIIC, for a total of 195 isolates (see Additional file 1 for extra information). Only *sigB *and *actA *sequence data had previously been reported for these 11 lineage III isolates [6]. Glycerol stocks of the isolates stored at -80°C were revived on BHI-agar plates and liquid cultures obtained from single colonies were used to make lysates.

### Genes Sequenced and MLST analysis

Partial DNA sequences of seven genes including four housekeeping genes (*gap*, *prs*, *purM *and *ribC*), two virulence genes (*inlA *and *actA*), and one stress response gene (*sigB*) were used for our analyses; a detailed description of the function of these genes and their position on the chromosome can be found in Nightingale *et al*. [2]. Sequencing of these seven loci for isolates without previously reported sequence information was performed as detailed by Nightingale *et al*. [2].

Assignment of sequence types (STs) and allelic types of the individual genes was performed using DNAsp 4.10.9 [19] as detailed by Nightingale *et al*. [2]. Previously reported STs and allelic types were numbered to be consistent with those reported by Nightingale *et al*. [2]; STs and allelic types not previously encountered were given new identification numbers. Isolate information and all sequence data used in this study are available in Pathogen Tracker http://www.pathogentracker.net. Sequences and alignments have also been deposited in Genbank (*actA *accession nr EU497055 to EU497238 and EU847029 to EU847039, *gap *accession nr EU520659 to EU520842 and EU847040 to EU847050, *inlA *accession nr EU520843 to EU521026 and EU847051 to EU847061, *prs *accession nr EU521395 to EU521578 and DQ347729 to DQ347720, *purM *accession nr EU525662 to EU525845 and DQ347741 to DQ347732, *ribC *accession nr EU521027 to EU521210 and DQ347753 to DQ347744, *sigB *accession nr EU521211 to EU521394 and EU847062 to EU847073).

### Descriptive analysis of sequence data

DNAsp 4.10.9 [19] was used to calculate the average pair-wise nucleotide difference per site (π), the average pairwise nucleotide difference per sequence (*k*), the number of polymorphic sites, the number of mutations, the number of alleles, the GC content, Tajima's *D *(test for neutrality of the data [20]), the number of synonymous and non-synonymous mutations, and the rate of non-synonymous to synonymous changes with a Jukes-Cantor correction. All calculations were performed for all isolates, and separately for the isolates belonging to lineages I and II. Separate analyses were not performed for the lineage III isolates due to the small number of isolates.

### Phylogenetic analysis

Phylogenetic relationships between the individual sequence types were inferred using ClonalFrame v1.1 [14]. This software is based on a model of genetic diversification that accounts for the way recombination occurs in bacterial populations. This enables the inference of phylogenetic relationships based on sequence data of multiple MLST loci, even if they are partly incongruent due to recombination. Besides the genealogy of the sample, ClonalFrame also infers when and where recombination events took place in the evolutionary history of the sample and estimates population-wide evolutionary parameters (e.g., mutation rate; recombination rate). Sequence data of the 92 unique STs identified among the 195 isolates were input into ClonalFrame and default values were used for all options. Five independent ClonalFrame runs were performed, each consisting of 300,000 iterations. The first 100,000 iterations in each run were discarded, and the phylogeny and additional model parameters were sampled every 100 generations in the last 200,000 iterations; thus, each run produced a sample of size 2,000 from the posterior. The convergence of the Markov Chain Monte Carlo (MCMC) in the different runs was judged satisfactory based on the Gelman-Rubin test [21] as implemented in the ClonalFrame GUI, and using Tracer v1.4 (available from A. Rambaut and A. J. Drummond at http://beast.bio.ed.ac.uk/). The samples from the five different runs were then concatenated for further analysis, resulting in a sample of size 10,000 from the posterior. The genealogies for our sample population were summarized in two complementary ways: a 95% majority rule consensus tree [22] constructed using the ClonalFrame GUI, and a consensus network built using Splitstree [23].

### Recombination and recombination rate

Sawyer's test of recombination was performed using GENECONV version 1.81a [24]. Analyses were performed using separate alignments for each of the seven loci; alignments were constructed using one sequence for each unique allelic type. The default settings for GENECONV were used and the number of recombinant events was inferred from the output as described in Nightingale *et al*. [2].

Recombination events were also assessed using ClonalFrame v1.1, assuming that a posterior probability of import above 95% is conclusive evidence for the occurrence of a recombination event. The recombination rates within lineages I and II were also inferred using ClonalFrame v1.1. For this purpose ClonalFrame was run independently using separate data sets that only included STs that grouped into lineage I and only the STs that grouped into lineage II. Because these datasets are smaller than the combined dataset for all lineages, we did not attempt to jointly infer the values of the mutation rate (θ) and the mean tract length of imported sequence fragments (δ). Instead, we set δ equal to the mean value inferred by the analyses of the whole dataset (δ = 122 bp), and we used fixed mutation rates (θ) for the individual lineages (i.e., θ = 91 for lineage I and θ = 60 for lineage II corresponding to the average value of θ obtained from single runs with 300,000 iterations). Two complementary measures of the recombination rate were calculated: ρ/θ which measures the relative frequency of occurrence of recombination and mutation in the history of the lineage [25], and r/m which measures the relative impact of recombination and mutation in the genetic diversification of the lineage [26].

To assess the influence of recombination on the inference of the phylogeny and particularly the branch lengths, five individual ClonalFrame analyses (100,000 burn-in iterations plus 200,000 sampling iterations) were performed without allowing for recombination (i.e., the recombination rate ρ was set equal to zero).

### Population History

To infer the population history of lineage I and lineage II we used the external/internal branch length ratio test [27] as implemented in the ClonalFrame GUI. This test calculates the ratio of the sum of the external branches (the ones that connect a leaf of the tree) to the sum of the internal branches for each tree in the posterior. The distribution of these ratios is than compared to the distribution of the external/internal branch length ratio as expected under the coalescent model [28]. If the distribution of this ratio is significantly larger than expected this means that the genealogy is unexpectedly 'star-like', which is consistent with a recent expansion of the effective population size, either due to a population bottleneck or a selective sweep.

### STRUCTURE analysis

To infer the ancestry of the lineage I and lineage II STs in our dataset, we performed an analysis using the linkage model of the program STRUCTURE [17,29]. This software assumes that the observed data is derived from K ancestral subpopulations. In the linkage model, the observed sequences are assumed to be made of blocks, each of which is inherited from one of the ancestral subpopulations. The program therefore infers for each site of each sequence its posterior probability of deriving from any of the K ancestral subpopulations, and by averaging these probabilities over all sites, we get the average proportion of genetic material derived from each ancestral subpopulation by each individual. To estimate K (the number of ancestral subpopulations), the probability of observing the data given a certain value of K (Pr(X|K)) was calculated for values of K ranging from 1 to 10 (three replicates per value of K). These values were estimated based on relatively short runs of STRUCTURE (20,000 burn-in iterations and 40,000 sampling iterations). STRUCTURE calculates a heuristic estimate of Pr(X|K) after each individual analysis. The value of K that maximized Pr(X|K) was then used for five longer runs (20,000 burn-in iterations and 100,000 sampling iterations).

## Results

### Descriptive analysis of sequence data

The 195 isolates characterized were classified into 92 unique sequence types (STs; see Additional file 1 for all STs and allelic types) whereas a previous analysis of 120 *L. monocytogenes *isolates from human and animal clinical cases and foods (Nightingale *et al*. [2]) only revealed 52 STs. The addition of sequences for 75 isolates from other sources thus resulted in a considerably more diverse data set for our population genetics analyses. The increase in STs is mainly caused by an increase in lineage II isolates; the 45 additional lineage II isolates contributed 26 new STs.

The genetic diversity of the seven loci sequenced ranged from π = 0.0044 (*gap*) to π = 0.0669 (*purM*) with the number of allelic types ranging from 18 (*gap*) to 50 (*inlA*) (Table 1). When the different parameters were calculated separately for isolates in lineages I and II, the genetic diversity in each lineage was typically considerably lower as compared to the overall genetic diversity (e.g., for *actA *π_all _= 0.0558, while π_linI _= 0.0058 and π_linII _= 0.0064); the only exception being *gap *(Table 1). One locus (*ribC*) showed a significant positive value for Tajima's D (Table 1) when the data for all lineages were analyzed, suggesting a divided population structure. On the other hand, *prs *showed a significantly negative value of Tajima's D in lineage II (-2.02, *P *< 0.05), which is indicative of a bottleneck or selective sweep [30,31]. For lineage II, Tajima's D was also negative for *sigB *(-1.69), however this value was not significant (*P *> 0.05).

**Table 1:** Descriptive analysis of nucleotide sequence data

All Isolates (n = 195)	Length of sequenced region (in bp)	No. of Poly-morphisms	No. of Mutations	No. of Alleles	GC content	π/site^*b*^	k^*c*^	Tajima's D^*d*^	Syn.^*e*^	Nonsyn.^*e*^	*dN/dS*^*f*^
*actA*	561	138	156	46	38.40	0.0593	33.26	0.78	59	70	0.32
*gap*	569	16	16	18	40.00	0.0044	2.50	-0.22	13	3	0.02
*inlA*	771	105	109	50	40.90	0.0264	18.90	0.04	64	44	0.10
*prs*	633	57	58	21	40.90	0.0209	13.25	1.01	58	0	n/a
*purM*	714	161	185	49	42.00	0.0669	47.74	1.61	133	26	0.05
*ribC*	639	121	131	34	39.60	0.0632	40.41	**2.51**	117	14	0.02
*sigB*	666	75	82	22	38.70	0.0251	16.63	0.57	76	3	0.02

**Lineage I ** **(n = 87)**											
*actA*	561	15	15	15	38.90	0.0058	3.24	0.25	6	9	1.06
*gap*	569	2	2	3	40.10	0.0009	0.54	0.55	2	0	n/a
*inlA*	771	17	17	12	41.00	0.0043	3.07	-0.26	6	11	0.32
*prs*	633	8	8	5	40.70	0.0018	1.14	-0.70	8	0	n/a
*purM*	714	10	10	13	42.40	0.0034	2.41	0.56	7	3	0.02
*ribC*	639	18	18	8	39.00	0.0061	3.91	0.27	15	3	0.06
*sigB*	666	9	9	8	38.60	0.0035	2.30	0.73	9	0	n/a

**Lineage II ** **(n = 93)**											
*actA*	561	19	20	17	37.90	0.0064	3.58	-0.25	11	9	0.42
*gap*	569	11	11	12	39.80	0.0050	2.85	0.85	8	3	0.04
*inlA*	771	54	54	25	41.30	0.0227	17.72	2.04	34	20	0.12
*prs*	633	18	18	4	41.10	0.0017	1.05	**-2.02**	18	0	n/a
*purM*	714	128	140	22	41.60	0.0603	43.05	1.91	108	26	0.08
*ribC*	639	86	88	16	40.20	0.0417	26.66	1.80	80	8	0.02
*sigB*	666	27	27	4	38.60	0.0035	2.35	-1.69	26	1	0.01

^a ^The total of 195 includes 15 lineage III isolates, which were not analyzed separately.

^b ^Average pairwise nucleotide difference per site.

^c ^Average pairwise nucleotide differences per sequence.

^d ^Tajima's D values significantly different from 0 (indicating deviation from standard neutral model) are marked in bold type.

^e ^Syn., number of synonymous mutations; Nonsyn., number of nonsynonymous mutations.

^f ^The dN/dS ratio was determined only when some nonsynonymous mutations were observed.

### Phylogenetic analysis

**Figure 1:**
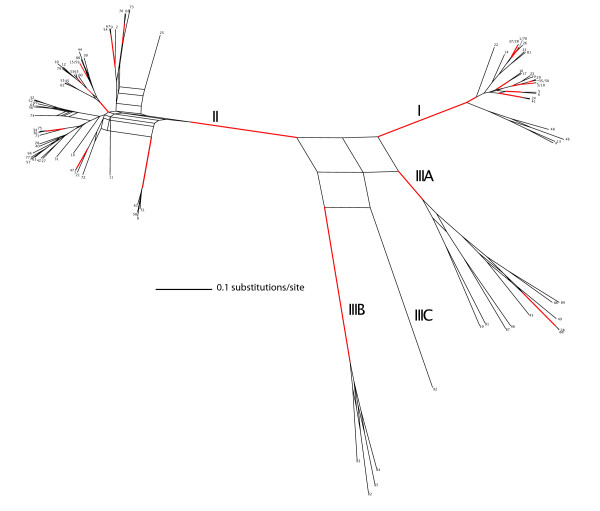
**Unrooted consensus network based on 10,000 phylograms obtained from ClonalFrame analyses of the individual sequence types**. Genealogical inference was performed for all 92 unique STs using ClonalFrame as described in the Methods. A consensus network was built using Splitstree [23]. Reticulate relationships were found in at least 20% of the trees and indicate phylogenetic uncertainty. Branches supported by a posterior probability of more than 95% are colored in red. Leaves are labeled with ST designations.

The consensus network tree based on the combined output of all five ClonalFrame runs (10,000 trees) shows that the clades representing lineage I and lineage II receive significant support (>95% posterior probability) (Figure 1). The lineage IIIA and IIIB isolates each form separate significantly supported clades (>95% posterior probability). The analysis is inconclusive about the monophyly of lineage III or the phylogenetic placement of lineage IIIC. Within lineage I, significant support was found for a subdivision between STs 4, 13, 46 and 48 and the remainder of the lineage I isolates. Lineage II contains several significantly supported clades of closely related STs.

### STRUCTURE analysis

**Figure 2:**
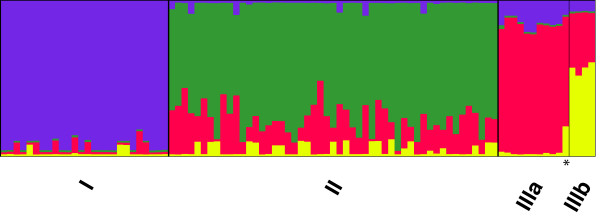
**Mixture of ancestry of the different STs as inferred by STRUCTURE**. Proportions of ancestry from ancestral lineage I (purple), ancestral lineage II (green), ancestral lineage IIIA (red) and ancestral IIIB subpopulations (yellow) as inferred by STRUCTURE assuming K = 4 ancestral subpopulations. The asterisk marks a lineage IIIC isolate. Each vertical line represents an individual sequence type and is colored according to the inferred proportion of single nucleotide alleles that were derived from one of the ancestral subpopulations. This bar plot was created with the DISTRUCT software [52].

Multiple STRUCTURE runs with different values of K (the number of ancestral subpopulations) showed that the probability of the data given K was maximal at K = 4. For simplicity we will call the ancestral populations with the highest proportion of lineage I, II, IIIA and IIIB isolates the 'ancestral' populations of the respective lineages [32]. Lineage I forms a genetically homogeneous group with an average of 93% of genetic material descended from the ancestral lineage I population (Figure 2). In contrast, only an average of 74% of the genetic material found in lineage II isolates is descended from the ancestral lineage II population (Figure 2), whereas 20% of it came from the ancestral lineage IIIA population. In particular, 14 STs in lineage II had an exceptionally low proportion (between 50 and 66%) of genetic material from the ancestral lineage II population. In these STs, up to 47% of the material came from the ancestral lineage III population. Due to the small number of lineage III isolates in our dataset (n = 15), we did not explore the population structure of lineages IIIA, B, and C. Rather, lineage III isolates were only included in the STRUCTURE analyses to estimate the proportion of lineage I and II genetic material that was obtained from the ancestral, lineage III populations.

### Population history

**Figure 3:**
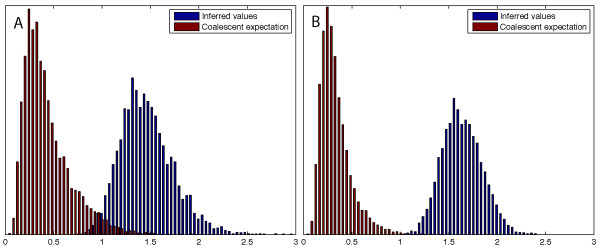
**Distribution of the Interior/exterior branch length ratio of trees resulting from ClonalFrame analysis of lineage I (A) and II (B) as compared to trees simulated under the coalescent model**. Both lineages show a higher internal/external branch length ratio (lineage I 1.50, *P *= 0.005; lineage II 1.64, *P *= 0.00002) than expected under the coalescent model, which is indicative of a bottleneck event during the population history.

The internal/external branch length ratio test showed that both recombination corrected genealogies of lineage I and lineage II exhibit a significantly higher internal/external branch length ratio (lineage I: 1.50, *P *= 0.005; lineage II: 1.64, *P *= 0.00002) than expected under the coalescent model (see Figure 3). This clearly indicates that the contemporary population in both lineages experienced a recent expansion of the effective population size, which is consistent with a population bottleneck.

### Recombination events

The majority of the 17 recombination events found by the Sawyer's test was found in the house-keeping genes *purM *(4 events) and *ribC *(8 events) (Table 2). The average tract length of recombination imports inferred by the Sawyer's test was 302 bp for *inlA*, 114 bp for *purM *and 192 for *ribC*. ClonalFrame also inferred a high number of recombination events in *purM *(13 events) and *ribC *(10 events), but in contrast to the Sawyer's test it inferred a high number of recombination events in the virulence genes *actA *(11 events, only one event according to the Sawyer's test) and *inlA *(16 events, only three events according to the Sawyer's test) (Table 3). The number of events inferred by ClonalFrame may be higher because it detects recombination events in a phylogenetic framework, which allows the detection of recombination events involving the entire length of the locus as well as events involving only parts of the locus. Closer examination of the results of both tests revealed that the majority of the recombination events in *actA *involved an 80 bp region on the 5'-end of the locus. The average tract length of recombination imports (δ) for all loci inferred from the combined ClonalFrame runs was 126 bp with a 95% credibility interval of 100 to 154 bp. While ClonalFrame may have a tendency to underestimate the tract length [14], this result is in fairly good agreement with the short lengths of recombinant fragments inferred by Sawyer's test.

**Table 2:** Summary of recombination analysis using Sawyers test (GENECONV)

	Global inner recombination^*a*^		Global inner events						
		
			Within lineages			Between lineages			
	Sim. *P *value^*b*^	Fragments^*c *^(events^*d*^)	I	II	III	I/II	I/III	II/III	multiple^*e*^

*actA*	0.0031*	19 (1 event)	0	0	0	0	0	1	0
*gap*	0.8650	0 (0 events)	0	0	0	0	0	0	0
*inlA*	<0.0001*	26 (3 events)	0	1	0	0	0	1	1 (II/II and I/II)
*prs*	0.0043*	3(1 event)	0	0	1	0	0	0	0
*purM*	<0.0001*	141 (4 events)	0	3	0	0	0	0	1 (II/II and II/III)
*ribC*	<0.0001*	68 (8 events)	1	2	0	2	0	0	3 (II/II and II/III)
*sigB*	0.0735	0 (0 events)	0	0	0	0	0	0	0

total		226 (17 events)	1	6	1	2	0	2	5

^a^*"*Global inner recombination" denotes recombination between ancestors of sequences in alignment; no significant support was found for recombination events involving an ancestor outside the alignment or events obscured by subsequent mutations (i.e., "Global outer fragments").

^b ^Sim. *P *value, simulated *P *value. *P *values indicating statistically significant (*P *< 0.05) evidence for recombination events are marked with asterisks.

^c ^Number of segments of alignment sufficiently similar to imply recombination.

^d ^Group of fragments linked to the same 5' and/or 3' breakpoints were classified as a single recombination event (as described by Nightingale et al., 2005).

^e ^"Multiple" refers to global inner recombination events that involved significant fragments between and within lineages.

**Table 3:** Recombination events inferred by ClonalFrame^1^

	No. of recombination events in			
	
Locus	Lineage I	Lineage II	Lineage III	Total
*actA*	0	9	2	11
*gap*	0	0	0	0
*inlA*	0	16	0	16
*prs*	2	3	1	6
*purM*	1	12	0	13
*ribC*	2	8	0	10
*sigB*	0	1	0	1

Total	5	49	3	57

^1 ^Only recombination events with a posterior probability of 95% or higher were counted

### Overall effect of recombination

To compare the rate of recombination between lineage I and II, ClonalFrame analyses were run separately on isolates representing each lineage (Table 4). We found a mean value for ρ (the recombination rate times two) of 11.4 (with credibility interval [2.2–21.8]) for lineage I, and of 42.6 (C.I. [29.3–56.3]) for lineage II. The relative frequency of occurrence of recombination versus mutation (ρ/θ) was 0.13 (C.I. [0.03–0.23]) for lineage I, and 0.71 (C.I. [0.49–0.94]) for lineage II. The relative effect of recombination versus point mutation (r/m) was 0.66 (C.I. [0.24–1.19]) for lineage I and 4.42 (C.I. [3.04–5.89]) for lineage II. Both the frequency and effect of recombination was therefore estimated to be about six times higher in lineage II than in lineage I, and the non-overlapping of the credibility intervals between lineage I and II strains for all parameters indicated that these differences are statistically significant.

**Table 4:** Recombination rates inferred by ClonalFrame analysis.

		ρ^a^	r/m^b^	ρ/θ^c^
Lineage I	Run 1	10.75 (3.25–22.83)	0.64 (0.27–1.28)	0.12 (0.04–0.25)
	Run 2	11.48 (4.27–22.77)	0.69 (0.29–1.27)	0.13 (0.05–0.25)
	Run 3	10.86 (2.59–21.00)	0.64 (0.26–1.21)	0.12 (0.03–0.23)
	Run 4	12.32 (3.35–27.06)	0.67 (0.27–1.25)	0.14 (0.04–0.30)
	Run 5	12.03 (4.19–22.55)	0.69 (0.28–1.26)	0.13 (0.05–0.25)

	All runs combined	11.4 (2.17–21.76)	0.66 (0.24–1.19)	0.13 (0.03–0.23)

Lineage II	Run 1	41.47 (30.32–56.30)	4.30 (3.17–5.76)	0.69 (0.51–0.94)
	Run 2	42.07 (29.89–56.13)	4.24 (3.00–5.79)	0.70 (0.50–0.94)
	Run 3	43.28 (31.86–58.83)	4.61 (3.35–6.17)	0.72 (0.53–0.95)
	Run 4	41.87 (29.93–55.59)	4.35 (3.12–5.86)	0.70 (0.50–0.93)
	Run 5	42.46 (28.77–61.04)	4.43 (3.02–6.26)	0.71 (0.48–1.02)

	All runs combined	42.65 (29.27–56.30)	4.42 (3.04–5.89)	0.71 (0.49–0.94)

Mean value of the parameters with the 95% credibility interval given in brackets.

^a ^Recombination rate

^b ^Relative impact of recombination as compared to point mutation in the genetic diversification of the lineage

^c ^Relative frequency of occurrence of recombination as compared to point mutation in the history of the lineage

**Figure 4:**
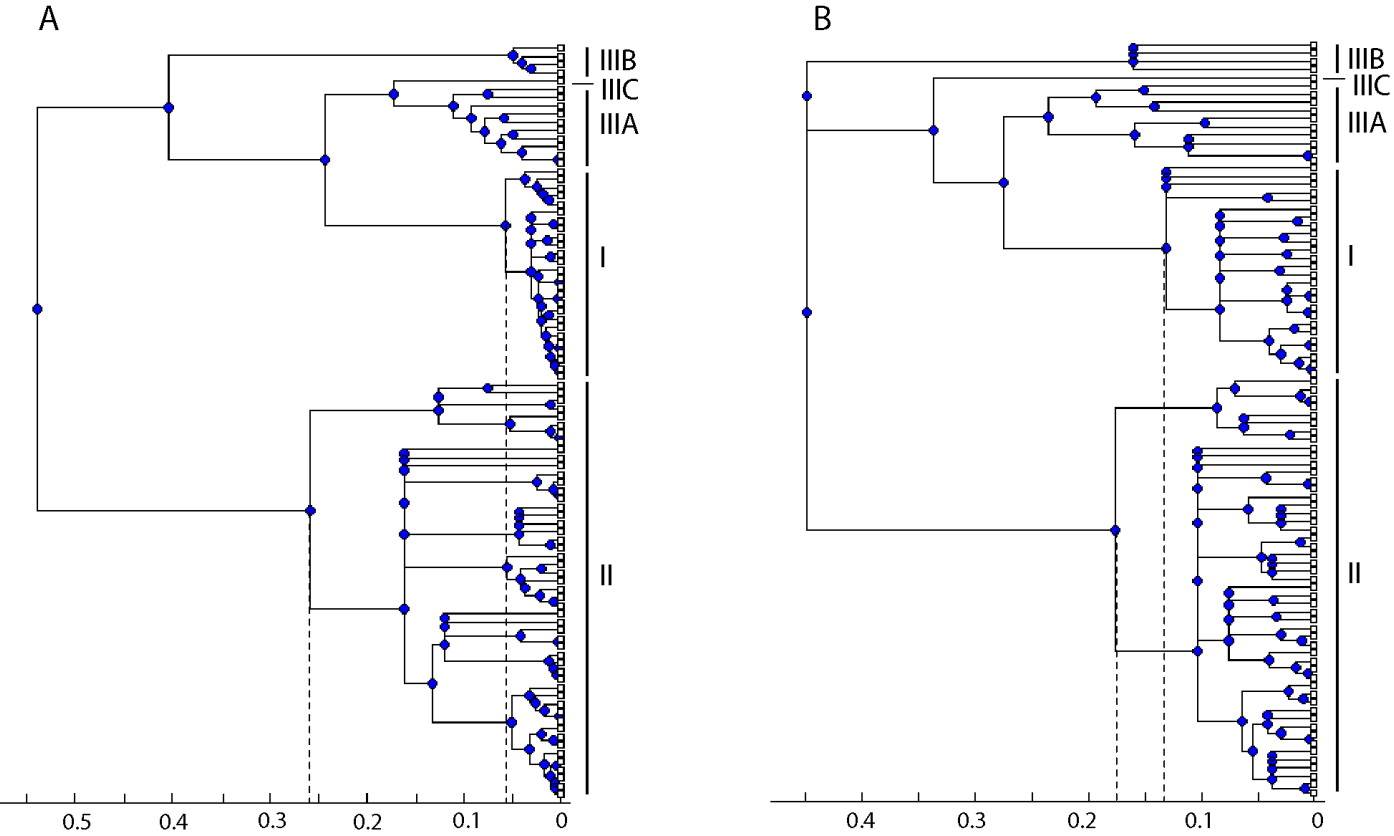
**Phylogenies inferred by ClonalFrame without (A) and with (B) correction for recombination**. The phylogram (A) shows a 50% majority-rule consensus tree based on ClonalFrame output (see Methods section) for all 92 unique STs and ignoring the role of recombination. The phylogram (B) is the same, but recombination was taken into account in the model of genetic diversification. The rulers indicate the time in coalescent units. Dashed grey lines show the estimated time to the most recent common ancestors of lineage I and II.

The influence of recombination on the phylogenetic inference of *L. monocytogenes *was clearly demonstrated by comparison of ClonalFrame results with and without correction for recombination (Figure 4). The phylogram based on the analysis without correction for recombination (Figure 4a) suggested a time to the most recent common ancestor (TMRCA) of lineage I that is clearly smaller than the TMRCA of lineage II, whereas a phylogram based on the analysis with correction for recombination (Figure 4b) revealed that the TMRCA of lineages I and II are similar. This indicates that lineage I and II appeared at approximately the same time, and that the higher genetic variability of lineage II can be attributed to its higher recombination rate.

## Discussion

The different tests and analyses we used all show that recombination is more prevalent in *L. monocytogenes *lineage II than in lineage I. The Bayesian inference of the relative recombination rate (ρ/θ) for both lineages shows that the relative recombination rate in lineage II is approximately six times higher than in lineage I (Table 4). Comparison of the values of ρ/θ with those that have been previously calculated for other species or groups of related species of bacteria (Table 5) shows that the recombination rate in lineage II is higher to that inferred for the *Bacillus anthracis/cereus*-clade [14], but still relatively low in comparison to pathogens such as *Neisseria meningitidis *[33]*Streptococcus pneumoniae *[34] or *Clostridium perfringens *[35]. While the higher genetic diversity among *L. monocytogenes *lineage II strains compared to lineage I strains has previously been reported [36,37], one other recent study [38] found no clear differences in recombination rates between *L. monocytogenes *lineage I and II isolates. By contrast, our data reported here indicate that the higher diversity among lineage II strains is exclusively due to a higher recombination rate in lineage II strains, and in particular to a large number of imports from lineage IIIA.

**Table 5:** Relative recombination rates found in other bacteria

Taxon	ρ/θ^a^	Reference
*Bacillus *(*anthracis/cereus *clade)	0.2–0.5	Didelot & Falush 2007^b^
*Listeria monocytogenes *Lineage I	0.03–0.23	this study^b^
*Listeria monocytogenes *Lineage II	0.49–0.94	this study^b^
*Streptococcus pneumoniae*	2.1	Fraser *et al*. 2005^c^
*Staphylococcus aureus*	0.11	Fraser *et al*. 2005^c^
*Clostridium perfringens*	3.2	Rooney *et al*. 2006^c^
*Neisseria meningitis*	1.1	Fraser *et al*. 2005^c^

^a ^Relative recombination rate expressed as the relative frequency of occurrence of recombination as compared to point mutation in the history of the lineage

^b ^Estimate obtained by Bayesian inference

^c ^Estimate obtained by Maximum likelihood

While both our data reported here and the data reported by Ragon et al. [38] are consistent with regard to the relative contribution of recombination and point mutations to the diversity of lineage I strains (r/m rates for nucleotides were between 0.6 and 0.7 in both studies), our data indicate a much higher r/m ratio for lineage II strains (r/m = 4.42) as compared to the r/m rate reported for linage II strains by Ragon et al. (r/m = 0.47) [38]. These differences in the relative recombination rates for lineage I and II strains between our study and the Ragon et al. [38] study are likely related to differences in the selection of isolates and target genes for MLST between these two studies. While the study of Ragon *et al*. [38] is heavily biased towards human clinical isolates (75%) with limited representation of animal (7%), food (3%) and environmental isolates (3%), 69% of the isolates in our study were from sources other than human clinical cases. The higher source diversity represented among our isolates, including use of a considerable number of environmental isolates that may be more likely exposed to donors of genetic material, may explain the increased frequency of recombination among the lineage II isolates characterized here. Ragon *et al*. [38] also used seven housekeeping genes, which are assumed to be under negative selection and subject to less homologues recombination, while our study used four housekeeping genes as well as two genes involved in virulence (*actA *and *inlA*) and one stress response gene (*sigB*). Interestingly, we identified recombination events in both virulence as well as housekeeping genes, consistent with a comparative genomic study by Orsi et al. [16], who found that close to 50% of the 2267 genes found in the *L. monocytogenes*/*L. innocua *core genome show evidence for recombination and that recombination is more frequent in lineage II than lineage I strains based on evaluation of 40 randomly selected genes. While the loci used by Ragon et al. [38] are thus ideal for intraspecific phylogenetic reconstruction, due to their low recombination rate, these genes are not necessarily representative for the recombination rate throughout the whole genome. The loci used in our study may be biased towards recombining loci and therefore lead to over-estimation of recombination rates, however the nearly identical r/m rates for lineage I isolates reported here and by Ragon et al. [38], suggest that selection of loci has a limited effect on estimates of recombination rate among lineage I strains. While multiple studies (e.g., Meinersmann *et al*. [36], Orsi *et al*. [16], etc.) support that recombination is frequent among lineage II strains, clearly some genes (e.g., those selected by Ragon et al. [38]) will show limited recombination in both lineages. An unbiased estimate of the contributions of recombination to diversity among bacteria will thus require future genome wide studies, such as those reported by Orsi et al. [16], and Lefebure and Stanhope [39]. Combined with an initial genome wide study already published (e.g., Orsi et al. [16]), our study shows that phylogenetic lineages, even within a given bacterial species, can differ significantly in their recombination and mutation rates. Similar findings have previously been reported for different species within a given genus. For example Lefébure and Stanhope [39], found that a much higher percentage (37%) of genes in the *Streptococcus *core-genome of *Streptococcus pyogenes *show evidence for recombination compared to *Streptococcus agalactiae *(18%). These results illustrate the need to develop new inferential tools that account for the possibility of variations in the recombination rate from one lineage to another.

Mutation hotspots, short stretches of sequence that evolve at a higher mutation rate than their flanking region, have been shown to cause homoplasy that can be incorrectly identified as being caused by recombination [40]. One stretch of sequence in our dataset, the first 80 bp of the *actA *locus, is potentially a mutation hotspot. Both the Sawyer test and ClonalFrame identify this stretch of sequence as being involved in recombination. ClonalFrame inferred that this fragment was involved in 9 independent recombination events. It is highly unlikely that the same stretch of sequence is involved in that many import events and therefore we consider this a case of mutation hotspot related homoplasy. The first 80 bp of the *actA *locus contain two amino acid positions that have been proven to be under positive selection [2], which may explain why this stretch of sequence experiences an elevated mutation rate. The occurrence of this hotspot may influence the inference of the recombination rate and the average length of the recombination fragments, however its effect is limited because it only involves ca 20% of the events inferred by ClonalFrame.

Mechanisms for horizontal gene transfer in bacteria include conjugation, transduction and transformation [41]. Transduction and conjugation usually involve long imported fragments (several kb's to hundred's of kb's) [41], and are therefore unlikely to be responsible for the homologous recombination events in *L. monocytogenes *we observed. Considering the short size of the recombinant fragments identified, horizontal gene transfer in *L. monocytogenes *most likely occurs through transformation. This is supported by the observation that short import elements of only a couple of hundred bp have been observed in the naturally competent bacterium *Helicobacter pylori *[42]. While *L. monocytogenes *does not show evidence for competence under laboratory conditions generally used to induce competency [43], our observations suggest that *L. monocytogenes *and in particular lineage II isolates may be competent under certain environmental conditions. This hypothesis is further supported by the fact that *L. monocytogenes *possesses a large number of genes (e.g., *comC*, *comEA*, *comEB*), which, in *B. subtilis*, encode proteins important for transformation [43].

While most lineage I and II strains possess the ability to cause listeriosis, lineage I, the lineage with the lowest recombination rate, appears to be more virulent for humans [44]. In particular, lineage I strains have been linked to almost all major human listeriosis outbreaks and are responsible for the majority of sporadic listeriosis cases [45], show a higher infectious dose [44], and, on average, have a higher ability to spread inside human cells [46]. In contrast, Wirth *et al*. [9] proposed, based on data for *Escherichia coli*, that epidemic and virulent bacteria face an increased selective pressure for rapid diversification in response to host immune defenses, resulting in higher recombination rates. As *L. monocytogenes *is an opportunistic pathogen with a wide host range as well as a saprotroph found in many different environments, we propose that the high recombination rate in lineage II is not due to selective forces involved in its virulence. Rather recombination may be critical for lineage II to successfully compete and survive in a broad range of different environments, consistent with the observation that lineage II strains are typically found at higher levels than lineage I strains (e.g., in foods [44]) and are more common than lineage I strains in natural environments.

Our data suggest that both lineages I and II have been subject to a recent expansion in population size, which is obscured in lineage II by a high level of recombination. The question is if this recent expansion started from a small founder population that survived a major extinction event (a population bottleneck) or from a limited number of strains with a higher fitness as compared to related strains in the ancestral population (selective sweep). It is difficult to differentiate between these two scenarios, since they both leave the same population genetic signal in a contemporary population. While previous studies [36,37] proposed that only *L. monocytogenes *lineage I strains experienced a recent bottleneck, our analyses suggest that both lineages have been affected. Using ClonalFrame, which takes recombination into account when reconstructing a phylogeny, we showed that lineage I and II do not differ considerably in age (assuming that the mutation rate follows a molecular clock in both lineages). The external/internal branch length test shows significant support for a bottleneck scenario in lineages I and II. Additional support for a bottleneck in lineage II is found in a significant negative value of Tajima's D for *prs *and an almost significant negative value for *sigB*; a negative value of Tajima's D indicates that these loci did not evolve neutrally and is suggestive of a selective sweep or a population bottleneck [30,31]. This signature of a bottleneck event in lineage II is not apparent among the other genes studied as most of them (i.e., *actA*, *inlA*, *purM *and *ribC*) were subject to a considerable number of recombination events in lineage II. The occurrence of a most recent common ancestor around the same time in two independent lineages supports the scenario of a population bottleneck (as opposed to a selective sweep), since it implies a common cause for the reduction in population size, such as a climatological change or an alteration of their habitat. Meinersmann *et al*. [36] estimated the time to the most recent common ancestor of lineage I to be between one-half and one million years ago. This coincides with the first glaciation cycles of the Pleistocene [47], which induced a rapid change both in climate and biota. A decline of available hosts and/or a strong selective pressure for adaptation to colder temperatures could therefore be the cause for the population bottlenecks in both lineages. It is tempting to speculate that the fraction of the population that survived may have been adapted to growth and survival at low temperatures (a hallmark phenotypic characteristic of *L. monocytogenes *[48]) and/or to specific mammalian hosts that were available at that time.

Our data also support *L. monocytogenes *lineage I and lineage II should be considered distinct species-like evolutionary lineages, consistent with the conclusions reached by Ragon et al. [38] in their recent study. The results of our ClonalFrame and STRUCTURE analyses support the existence of lineage I and II as separate evolutionary lineages with little genetic material exchanged between them, which is in agreement with a number of previous studies [2,49]. The number of lineage III isolates in this study is too low to draw conclusions on the evolutionary status of this lineage. Though a well-supported subdivision was found within lineage I in the phylogenetic analysis, no evidence for a further subdivision of lineage I and lineage II could be found with the STRUCTURE analysis. The STRUCTURE analysis showed that 14 lineage II STs acquired less than 67% of their nucleotide alleles from the ancestral lineage II population. Wirth *et al*. [9] considered isolates with less than 2/3 overlap with one of the ancestral populations to belong to a hybrid group. The uncertainty in our phylogenetic inference (the reticulate relationships depicted in Figure 1) of the lineage II STs seems to be mainly caused by these 'hybrid' STs. Therefore lineage II could be considered a so-called fuzzy species [50], i.e., a species that has unclear boundaries because of its inherent ability to import genetic material from other species. A previous study [51] has shown that in the case of *Neisseria gonorrhoeae*, the apparent status as a fuzzy species was an artefact caused by sequencing of differentially amplified fragments from mixed non-viable historical cultures. In our case all sequences were obtained from viable cultures and started from single colonies in order to avoid mixed cultures. We can therefore be confident that lineage II truly represents a fuzzy species. Lineage I, on the other hand, seems to be a species with clearly defined species boundaries and few imports from an external origin.

## Conclusion

Our study not only adds to an emerging body of literature that supports the importance of recombination in the evolution of bacterial populations, but also shows that even closely related bacterial populations can differ considerably with regard to the contributions of homologous recombination to diversification and evolution. Our study further illustrates the challenges that lineage specific recombination provides for inference of the phylogeny and population history of bacterial populations. While *L. monocytogenes *lineage I has traditionally been seen as less divergent and more clonal than lineage II, our analyses clearly show that high diversity in lineage II is due to recombination and that both lineages went through a population bottleneck. Previous suggestions that only lineage I experienced a bottleneck appear to be an artifact of the high recombination rate in lineage II. While both lineages I and II seem to represent species-like lineages, lineage I appears to be a genetically isolated population with a low recombination rate, whereas lineage II seems to have more diffuse genetic boundaries due to a relatively high frequency of imports of genetic material from other lineages. While our data supports that lineage I and lineage II each constitute species from an evolutionary perspective and while the two lineages appear to differ in their virulence [44], they both are able to cause human listeriosis. From a practical perspective, it may therefore be best not to change theses lineages to an official rank of species.

## Authors' contributions

HCdB participated in the design of the study, performed the phylogenetic and statistical analyses, participated in the sequence alignment and drafted the manuscript. XD helped with the ClonalFrame analyses and helped to draft the manuscript. EDF performed the collection of the sequence data and participated in the sequence alignment. KN participated in the design of the study and helped to draft the manuscript. MW conceived of the study, and participated in its design and coordination and helped to draft the manuscript. All authors read and approved the final manuscript.

## Supplementary Material

Additional File 1 **Isolates.** This table contains the list of the isolates used in this study and additional information on the source of the isolates, their serotypes, ribotypes and sequence types. Click here for file
